# Determination of salt content in traditional and industrial Moroccan white bread by inductively coupled plasma mass spectrometry

**DOI:** 10.11604/pamj.2022.42.79.33961

**Published:** 2022-05-30

**Authors:** Meryem Bouhamida, Meryem Lazrak, Houria Lahmam, Sara Ait Lachguer, Yasmine Guennoun, Laila El Ammari, Abdelhakim Yahyane, Nour Eddine Elhaloui, Naima Saeid, Nada Benajiba, Radouan Saadi, Khalid El Kari, Radhouene Doggui, Ayoub Al Jawaldeh, Hasnae Benkirane, Hassan Aguenaou

**Affiliations:** 1 Ibn Tofaïl University, *Centre National de l'Énergie, des Sciences et Techniques Nucléaires (CNESTEN)*, Joint Research Unit in Nutrition, Health and Environment, Regional Designated Centres (RDC)-Nutrition African Regional Cooperative Agreement for Research, Development and Training Related to Nuclear Science and Technology (AFRA)/ International Atomic Energy Agency (IAEA), Laboratory Biology and Health, Kenitra, Morocco,; 2 Ministry of Health, Rabat, Morocco,; 3 Department of Basic Health Sciences, Deanship of Preparatory Year, Princess Nourah Bint Abdulrahman University, Riyadh, Saudi Arabia,; 4 Laboratory Division, National Center for Energy, Sciences and Nuclear Techniques, Rabat, Morocco,; 5 New Brunswick Medical Training Center (University of Sherbrooke), Pavillon J-Raymond-Frenette, Moncton University, Moncton, Canada,; 6 Nutrition, Non-Communicable Diseases and Mental Health Department, World Health Organization (WHO), Regional Office for the Eastern Mediterranean (EMRO), Nasr City, Cairo, Egypt

**Keywords:** Sodium, salt, bread, bakeries, inductively coupled plasma mass spectrometry (ICP-MS)

## Abstract

**Introduction:**

evaluating the sodium content of staple foods is essential for implementing a salt reduction strategy. In Morocco, bread is a major contributor to sodium intake. However, currently few studies have been carried out to assess the salt content in bread. Our study aimed to estimate the sodium and salt content of white bread available in artisanal and industrial bakeries in the twelve regions of Morocco.

**Methods:**

it is a cross-sectional study of the sodium content of white bread available for sale in artisanal and industrial bakeries in Morocco (N=120). Inductively coupled plasma mass spectrometry (ICP-MS) was used to quantify the sodium content of the bread. The percentage of samples meeting the recommendations and bread contribution to the daily salt intake was calculated.

**Results:**

the results of our study show that the mean levels of sodium and salt added to bread samples were 5.7 ± 1.5 g/Kg and 14.5 ± 3.7 g/Kg, respectively. With an average of 4.4 ± 0.5 g/Kg and 11.2 ± 1.2 g/Kg for artisanal bread and an average of 7.0 ± 0.8 g/Kg and 17.8 ± 2.1 g/Kg for industrial bread, respectively. Daily salt intake from bread consumption (500 g/d/person) is estimated at 5.6 g/d (52.8% of total salt intake) for artisanal bread and 8.9 g/d (84% of total salt intake) for industrial bread.

**Conclusion:**

bread salt content in Morocco exceeds the recommended threshold of the national federation of bakery and pastry and health authorities. Further efforts are necessary to increase knowledge and awareness of bakers and to teach them how to reduce salt content without affecting the flavor and the quality of their products.

## Introduction

Salt or table salt is widely used nowadays in food processing and production. It was discovered in 1807 by the British chemist and physicist Humphrey Davy [1]. For several million years, humans have eaten very small amounts of salt (0.1-0.5 g/day) as before the invention of the freezer and the refrigerator, it was mainly used as a food conservative [2]. Since it started being used in highly salted processed food, salt intake by humans worldwide is continuously increasing. According to Brown *et al*. (2009) daily salt consumption is ranging between 9-12 g/individual worldwide [3]. This amount is almost the double of World Health Organization´s (WHO) recommendation which is of 5 g/day/pr [4]. The pioneering intersalt study (1988) reported that an increase in salt intake by 6 g/d could lead to an increase in systolic blood pressure by 9 mm Hg over 30 years [5]. In addition, it might also directly increase the risk of stroke and coronary heart disease (CHD) [6]. Furthermore, it has been demonstrated that a reduction of 6 g/d in salt intake could reduce heart stroke incidence by 24% and CHD by 18% [7]. High salt intake is also associated with increased risk of other diseases such as renal diseases, stomach cancer, osteoporosis and obesity [8-11]. For example, the risk of obesity in adults will increase by 26% as a result of an increase in salt intake by 1 g/d (odds ratio, 1.26; 95% CI, 1.16-1.37; P<0.0001) [12]. In fine, globally, 3 million deaths were attributable to high sodium consumption, according to the “Global Burden of Disease” study [13].

In Morocco, the epidemiological and demographic transition is reflected in an increase in the burden of morbidity and mortality resulting from non-communicable diseases (NCDs). These diseases are currently the leading cause of death among all causes, with a prevalence of 80% deaths [14]. Indeed, according to Mozaffarian *et al*. (2014), high salt intake is the primary cause of high blood pressure [15], which is affecting more than a quarter (29.3%) of the Moroccan adult population [16]. According to WHO, about 75% of daily salt intake by humans is from processed foods [17]. This alarming amount is a logical consequence of the increased frequency of consumption of high salt content food namely fast food, processed meats, cheese, salty snacks or instant noodles [18,19]. Besides, the consumption of large quantities of food items such as bread and bakery products has also been indicated as a contributing factor to salt intake [20]. Another important source of salt in human intake is the added salt, for example broth and bouillon cubes or at the table (soy sauce, fish sauce and table salt). Subsequently, decreasing salt intake in modern populations' food habits is very challenging. With this regard, it becomes complicated to lower salt intake only through approaches targeting consumer attitudes and behaviour. This is even more difficult because the predominant salt sources are outside of their control [21]. Then, the population might be unaware of the actual consumed amount of salt [22].

In response to this global health problem, WHO developed the global action plan targeting a 30% reduction of the average salt intake by 2025, as one of nine targets to be achieved to reduce mortality from NCDs by 25% [23]. This plan includes many actions such as: i) Consumer awareness campaigns on benefits of salt intake reduction, providing a clear front-of-pack nutrition labelling to allow consumers to identify products with reduced salt content, and; ii) reformulate certain industrial food by reducing their salt content [24]. In fact, this latter has been demonstrated to be the most cost-effective measure due to the large quantities of high salt coming from processed foods [21,25]. Several countries have established voluntary or legislative programs to promote a progressive salt reduction to ensure a better consumer acceptance of new formulated foods items in addition to maintaining the same food safety and quality [26]. Previous studies found that reducing slowly and gradually the salt content in bread is not noticeable by consumers. According to El Ati *et al*. (2021) a 35% reduction in the salt content of white bread can be delivered without detection by Tunisian consumers [27]. In another study, a 52% salt reduction in bread did not lead to lower consumption of bread [28].

Food reformulation interventions in the Eastern Mediterranean Regional Office (EMRO) region have mainly focused on bread, the staple food in most EMRO countries [20]. Studies in the region have indeed shown that bread is one of the main contributors to salt / sodium intake in the diet [20,29-31]. Few countries like Iran, Jordan, Saudi Arabia, Kuwait and the United Arab Emirates have extended their food reformulation interventions to other food products, including dairy products, savory snacks, processed meats, etc. [32].

In the same optic, the Moroccan Government has developed a national multisectoral strategy for the prevention and control of NCDs 2019 - 2029 [33]. One of the targets related to NCDs set in this strategy is reducing by 10% of salt consumption among the Moroccan population by 2029. The stepwise survey showed that the Moroccan adults population consumes 10.6 g of salt per day [16]. Moreover, bread is an important component of the Moroccan diet as the daily average consumption is estimated at 500 g/individual/day [34]. Furthermore, wheat consumption in Morocco per capita is estimated at 173 kg annually, which is among the highest in the world (average world consumption is 152 kg) [35]. In addition, the bakery industry is a continuously growing market showing an important increase in sales of bakery product in Morocco passing from 827.4 US$ in 2010 to 1074.5 US$ in 2014 [36]. Bakery industries as an important factor in this strategy are endeavoring to reduce the salt content in their products [37,38]. Reducing the amount of salt added to bread could be an effective measure for the prevention and control of NCDs. However, currently few studies have assessed the sodium content in salt at the national level, especially in the EMRO where bread is a fundamental part of the local diet. Therefore, the current study aims to examine the salt content in artisanal and industrial bread from the 12 administrative regions of Morocco.

## Methods

**Figure 1:**
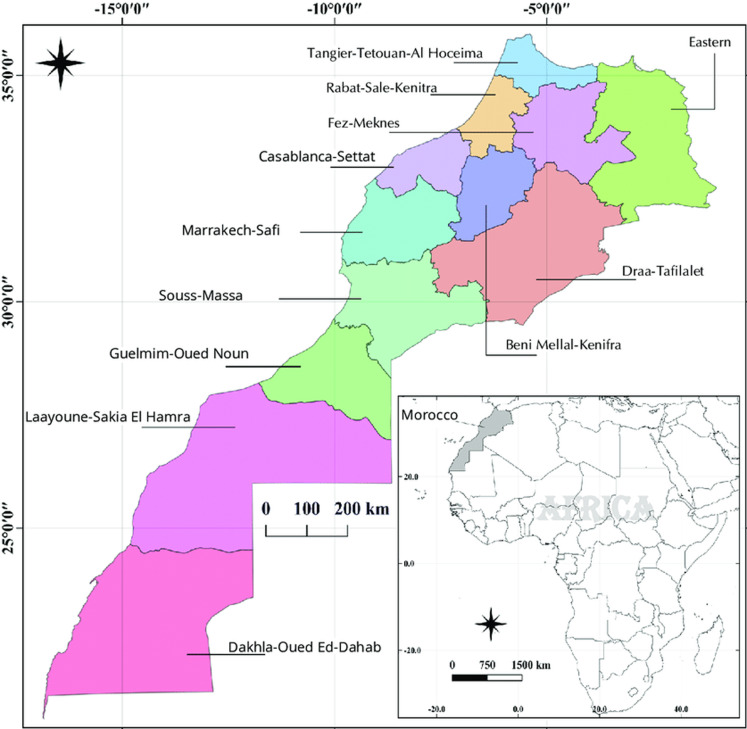
geographic map of Morocco

**Study design and data collection:** it is a cross-sectional study aiming to assess salt content in bread from different bakeries in the twelve regions of Morocco [39] (Figure 1). The collection of bread samples was conducted during 3 months from June to September 2020. One white bread sample (bread made from wheat flour from which the darker, coarser bran has been removed, retaining the starchy endosperm [40]) were collected from 10 different bakeries in each region. The 10 samples were consisting of 5 samples from artisanal bakeries and 5 from industrial bakeries. So, in total of 120 bread samples were collected and packaged in airtight plastic and labelled bags (one bag for each bakery) stored in cool and dry conditions and transported to the laboratory.

**Sample conditioning:** the bread samples were packaged, labelled and stored in separate airtight plastic bags at 15°C in the laboratory until use. The samples were analyzed to quantify the sodium content.

**Determination of sodium and moisture content:** the collected bread samples were first weighed and dried in an oven set at a temperature of 60°C for 24 h to determine the moisture content. Sodium analysis was performed with the inductively coupled plasma mass spectrometry (Xseries ICP-MS) after digestion of samples using microwave (Speed Wave 4) with nitric acid (HNO_3_). For this purpose, 250 mg of dry bread were weighed into microwave Teflon vessels (samples: triplicates; blank: single measurement; controls: duplicates) and dissolved in a mixture of 5 ml of nitric acid HNO_3_ (65% w/v) and 1 ml of hydrogen peroxide H_2_O_2_ (30% w/v). The digestion vessels were placed in the microwave for 45 minutes until the whole organic matter was completely digested. After completion of mineralization, digests were diluted appropriately (approximately 45 minutes), the mineralized samples were transferred into labeled, pre-weighted 50 ml polyethylene bottles, the vessels were rinsed 3 times with a few ml of nanopure water. Then, the mineralizates were completed gravimetrically to appropriate weight (50g), according to expected analyte concentration with nanopure water. After dilution, digests were aspirated to the plasma for the determination of metal contents by ICP-MS. The laboratory was equipped with a Thermo Xseries 2 quadrupole ICP-MS system, fitted with a Cetac 500 sample changer, and controlled by PlasmaLab operating software. After calculating the sodium content, the salt content has been calculated:

The standard reference material NIST 1567b (wheat flour, 6.71 ± 0.21 mg/Kg sodium) was analyzed at the beginning of each serie. The recovery was found to be satisfactory (97.5%).

**Calculation of the compliance with recommendations for the level of sodium (Na) in bread in Morocco:** the two specific guidelines for the maximum salt content of bread in Morocco were used as a reference. The first one is the Moroccan Federation of Bakeries which recommends adding the equivalent of 18 g of salt per kg of bread and the second one is the world health organization which recommends 5 g of salt per day (equivalent of 10 g of salt per kg of bread based on a daily consumption of 500 g/day of bread).

**Statistical analysis:** data were analyzed with the Statistical Package for the Social Sciences (SPSS) program version 22.0. In addition to descriptive statistics, one-sample t-test was run to establish differences between the types of bakeries, one-way ANOVA-test to establish differences between samples of each region and the paired samples t-test to establish differences before and after 10% reduction of salt content. Significance was set at p<0.05.

## Results

**Figure 2:**
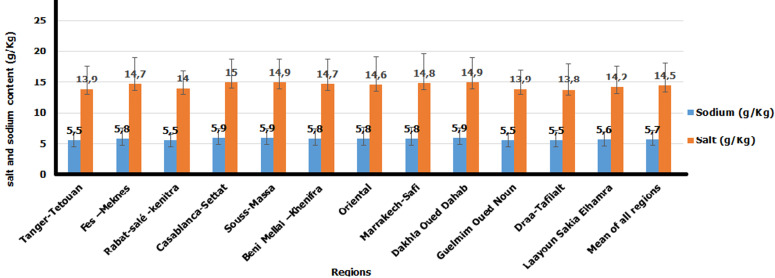
average sodium and salt added for the preparation of bread samples

**Average sodium and salt content in bread samples:** Figure 2 shows the average sodium and salt contents of bread samples consumed in Morocco. The average sodium and salt levels in the samples of bread from the 12 regions of Morocco were respectively 5.7 ± 1.5 g/kg and 14.5 ± 3.7 g/kg. The lowest salt content was found in Draa-Tafilalt (13.8 ± 4.2 g/Kg) and the highest in Casablanca-Settat Region (15.0 ± 3.7 g/kg). No difference statistically significant between regions was found.

**Figure 3:**
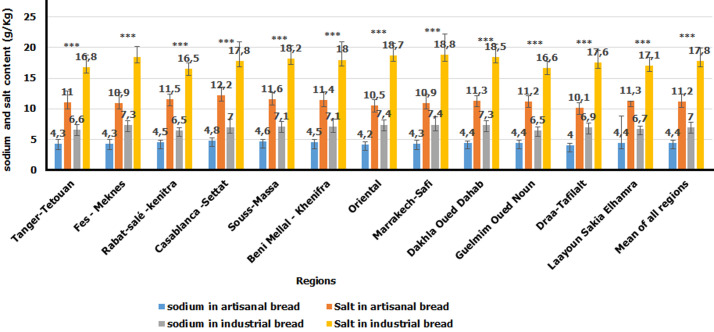
sodium and salt content in artisanal and industrial bread samples

**Sodium and salt content in artisanal and industrial bread samples:** the sodium and salt contents of the artisanal and industrial bread samples consumed in Morocco are shown in Figure 3. The average sodium and salt levels added to prepare artisanal bread samples were 4.4 ± 0.5 g/Kg and 11.2 ± 1.2 g/Kg, respectively. While the average contents of sodium and salt in the industrial samples were respectively 7.0 ± 0.8 g/Kg and 17.8 ± 2.1 g/Kg. Ranging between 6.5 ± 0.4 g/Kg and 16.5 ± 1.0 g/Kg in Rabat-Salé-Kénitra and 7.4 ± 1.3 and 18.8 ± 3.4 g/Kg in Marrakech-Safi. The comparison in term of salt content between the two types of bread has shown a difference statistically significant between the amounts of salt in artisanal and industrial bread (p <0.005).

**Figure 4:**
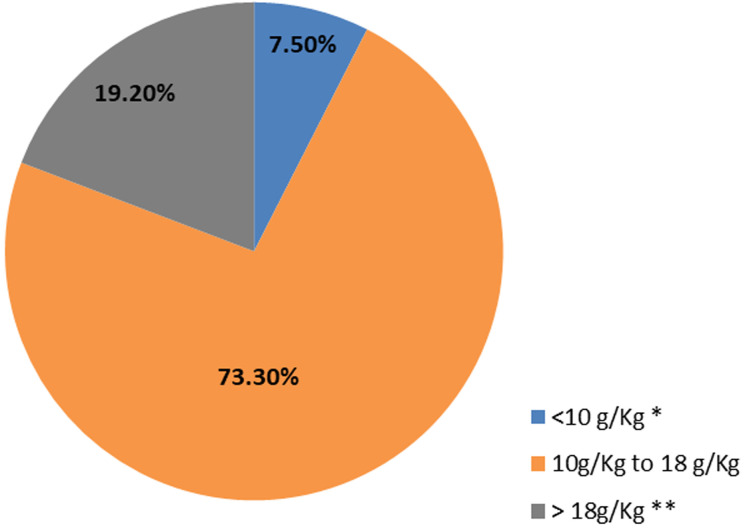
repartition of samples according to the recommendation of World Health Organization and the Moroccan Federation of Bakeries

**Percentage of samples respecting the recommendation of World Health Organization and the guidelines of the Moroccan Federation of Bakeries in term of salt adding:** Figure 4 represents the percentage of samples according to the recommendation of salt content of the world health organization and the Moroccan Federation of Bakeries guidelines. The results show that only 7.5% of the bread samples had a salt content lower than 10g per kg meeting the WHO recommendations. As the Moroccan Federation of Bakeries recommends adding the equivalent of no more than 18g of salt chloride per Kg of bread during the preparation of the white bread practically 19.2% of samples exceeded both recommendations.

### Contribution of bread´s salt in the total salt intake

**Contribution of artisanal bread´s salt in the total salt intake:** the estimation of the artisanal bread´s salt contribution (%) to the total salt intake (g/d/ person) is represented in Table 1. The average daily intake of salt from bread consumption (500 g/d/person) is 5.6 g/d varied between regions from 5.1 g/d in Draa-Tafilalt to 6.1 g/d in Casablanca-Settat which contribute to the total daily salt intake by 47.6% in Draa-Tafilalt to 57.5% in Casablanca-Settat with an average 52.8%.

**Table 1:** percentage of contribution of artisanal bread to the total salt intake

Region	Salt (g/Kg)	Daily salt intake from bread (500g/d/person*)	Percentage of bread contribution to total daily salt intake** (%)
Tangier-Tetouan	11.0 ± 1.9	5.5	51.9
Fes -Meknes	10.9 ± 1.7	5.5	51.4
Rabat-Salé-Kenitra	11.5 ± 0.9	5.8	54.2
Casablanca-Settat	12.2 ± 1.3	6.1	57.5
Souss-Massa	11.6 ± 1.0	5.8	54.7
Beni Mellal -Khenifra	11.4 ± 1.4	5.7	53.8
Oriental	10.5 ± 1.0	5.3	49.5
Marrakech-Safi	10.9 ± 1.5	5.5	51.4
Dakhla-Oued Dahab	11.3 ± 0.8	5.7	53.3
Guelmim-Oued Noun	11.2 ± 0.9	5.6	52.8
Draa-Tafilalt	10.1 ± 0.9	5.1	47.6
Laayoun-Sakia Elhamra	11.3 ± 1.1	5.7	53.3
Mean	11.2 ±1.2	5.6	52.8

* Bread consumption in Morocco (500g/d/ person); ** daily consumption of salt in Morocco 10.6 g/d/person

**Contribution of industrial bread to total daily salt intake:** estimations of industrial bread contribution (%) to total salt intake (g/d/person) are represented in Table 2. The daily intake of salt from bread consumption (500 g/d/person) varied between regions from 8.25 g/d in Rabat-Salé-Kenitra to 9.4 g/d in Marrakech-Safi. The percentage of industrial bread contribution to the total salt intake is 84% ranging from 88.7% in Rabat-Salé-Kenitra to 88.7% in Marrakech-Safi based on a 500 g daily bread intake in Morocco.

**Table 2:** percentage of contribution of industrial bread to the total salt intake

Region	Salt (g/Kg)	Estimated daily salt intake from bread (500g/d/ person*)	Percentage of bread contribution to total salt intake** (%)
Tangier-Tetouan	16.8 ± 2.0	8.4	79.2
Fes -Meknes	18.5 ± 1.7	9.25	87.3
Rabat-Salé-Kenitra	16.5 ± 1.0	8.25	77.8
Casablanca-Settat	17.8 ±3.1	8.9	84.0
Souss-Massa	18.2 ± 2.1	9.1	85.8
Beni Mellal -Khenifra	18.0 ± 2.9	9	84.9
Oriental	18.7 ± 2.0	9.35	88.2
Marrakech-Safi	18.8 ± 3.4	9.4	88.7
Dakhla-Oued Dahab	18.5 ± 2.0	9.25	87.3
Guelmim-Oued Noun	16.6 ±1.2	8.3	78.3
Draa-Tafilalt	17.6 ± 1.9	8.8	83.0
Laayoun-Sakia Elhamra	17.1 ± 1.3	8.55	80.7
Mean	17.8 ± 2.1	8.9	84.0

* Bread consumption in Morocco (500g/d/ person); **consumption of salt in Morocco 10.6 g/d/person

## Discussion

The current study is a part of the national multi-sectoral strategy (2019-2029) for the prevention and the control of NCDs among Moroccan population and the national program of nutrition. Thus, it aimed to quantify the salt content in artisanal and industrial bread from the twelve regions of Morocco. Results of the present study showed that the mean levels of sodium and salt added to the flour for the preparation of bread samples collected from the 12 regions of Morocco were 5.7 ± 1.5 g/Kg and 14.5 ± 3.7 g/Kg respectively. With an average of 4.4 ± 0.5 g/Kg and 11.2 ± 1.2 g/Kg for artisanal bakeries and an average of 7.0 ± 0.8 g/Kg and 17.8 ± 2.1 g/Kg for industrial bakeries with a statistically significant difference between artisanal and industrial bread (p<0.005). Daily salt intake from bread consumption (500 g/d/ person) varied considerably between the two types of bakeries, it is estimated at 5.6 g/d for artisanal bread and 8.9 for industrial bread. The contribution of bread to daily salt intake is 52.8% for artisanal bread and 84% for industrial bread based on a 500g daily bread intake in Morocco. Our results clearly demonstrate that eating bread alone is enough to provide sodium intake by Moroccan individual exceeding all these recommendations by at least 50%.

For comparison, a Nigerian study quantifying salt (sodium chloride) content of retail samples of white bread showed that the salt content varied extensively, ranging from 5.1 g/Kg (0.51%) to 18 g/Kg (1.8%). The average salt content was 13.6 g/Kg [41]. Another study quantifying the added salt in bread samples from 80 professional bakeries in Casablanca in Morocco showed that the average amount of added salt during the preparation of white bread is 17.42 ± 1.28 g/kg. This is the equivalent of a daily intake of 8 to 9 g of salt through bread alone. And that exceeds all recommendations [42]. According to a previous study on exploring the baker´s perspective with regard to their contribution to the implementation of the national strategy of salt reduction in Morocco. The bakers have declared that they usually add an average of 12.5 g/kg of salt to bread [43]. A salt analysis in bread from Croatian bakeries reported an average of 5 g of sodium/ Kg of bread [44]. In Mozambique Silva *et al*. (2015) indicated that a mean salt content of 11.4 g/Kg of bread (1.1% calculated on salt) [45]. In a Latin American country (Peru) salt content in bread was higher as it was 12 g/Kg of bread [46]. In Bosnia and Herzegovina, salt content was even higher as it found that the amounts ranged between 13 and 20 g/Kg of bread [47] and in Serbia it was ranging between 11 and 24 g/Kg of white bread [48]. A study in the Arab Region showed that the level of salt was also high, as in Kuwait 10.97 g/kg (1.1%), while Jordan produced the same flat bread at a level of 4.3 g/kg (0.43%). This study reported that the highest level of salt was found in the French type rounded thick bread produced in Tunisia 12.41 g/kg (1.24%) which contributed to 3.2 g of salt intake daily this represents 64% of the recommended salt intake level set by the WHO (<5 g/day/person) [20].

It is worth mentioning that one of nine targets in 2025 of the 66^th^ World Health Assembly endorsed by the WHO Global Action Plan for the Prevention and Control of NCDs 2013-2020 to achieve a 25% relative reduction in premature mortality from NCDs is 30% reduction in mean population salt intake by 2025. However, all these action plans and targets requires full collaboration with all the stakeholders, mainly food industry [26]. As such, bread as a staple food in Morocco contributes largely to dietary salt intake. And attention to reduce salt in bread has grown worldwide, with successful reports from several countries [37,49].

Indeed, previous studies showed that a reduction in salt content of up to 29% in bread is acceptable by consumers [26,37,49-53]. A Tunisian study on the feasibility of reducing salt in bread showed that a 35% gradual decrease in salt content was possible without detection by consumers, the salt concentration in bread was reduced from 17 ± 0.2 g/Kg at 11 ± 0.1 g/Kg (p<0.0001) [27]. Moreover, this level of reduction faced challenges due to the techno-functional and sensory roles of salt in bread [54]. Indeed, salt has essential functions that affect the quality of bread. Belz *et al*. (2012) reported that salt provides bread with sensory characteristics, controls the yeast growth and fermentation rate, improves the bread texture and extends the shelf life by reducing spoilage [55].

Several studies have shown that it is technically possible to reduce the amount of salt without affecting the consumer preference or sales. Indeed, it has been demonstrated that the salt content of bread can be reduced by 25% with no detection [49]. According to findings published recently by Guennoun *et al*. (2019), 21.8% of the Moroccan consumers agree to purchase bread with 23% salt reduction and 41.8% agree purchasing bread at 16% of salt reduction [52]. In addition, La Croix *et al*. (2015) reported that reducing sodium levels in bread up to 30% did not affect consumer liking or purchase intent of the products [56]. Another study showed that a salt reduction of 10 to 20% in whole bread does not affect the taste of the bread and could not be detected by tasters [57]. A study in Peru about the feasibility of reducing salt in bread showed that the introduction of bread with a 20% reduction in salt is feasible without affecting taste or bakery sales [45].

The implementation of clear monitoring approaches is crucial to demonstrate effectiveness, and to incentivize larger changes, especially for voluntary strategies [26]. In the EMRO Region, only six countries (Jordan, Saudi Arabia, Morocco, Oman, Qatar and Tunisia) have mechanisms established for monitoring sodium content in one or more food categories, and using laboratory analyzes. The latter is undoubtedly a very accurate method for determining salt level, but, given its cost, it may only cover a limited range of products rather than the entire food supply [58]. It may be necessary to conduct comprehensive surveys of salt levels in food products, based on validated product label data, an approach that can complement laboratory analysis of specific foods, to ensure that progress is made on a larger scale [59].

The present study is very informative and has many strengths. First and to our knowledge, it is the only research carried out so far in the 12 different regions of Morocco in order to assess salt concentration in bread on a national scale. It gave a first insight in response to the urgent need for information about salt content in bread. However, this study has some limitations in terms of convenience sampling of the bakeries meaning that generalization of the results is limited. Future studies are recommended to include more bakeries and all cities of each region to obtain a better insight into salt addition in these products. Additionally, it would be interesting to include information about the amounts of bread and bakery products consumed by the population in each region in order to estimate total salt consumption through this food item accurately.

## Conclusion

Bread salt content in Morocco exceeds the recommended threshold of the national federation of bakery and pastry and health authorities. Further efforts are necessary to increase knowledge and awareness of bakers and to teach them how to reduce salt content without affecting the flavor and the quality of their products especially for industrial bakers. The amount of salt added to bread should be standardized and an adequate legislation should be developed to guide bakers. It is strongly recommended to set an upper limit for the salt content of bread and to encourage consumers to reduce their consumption of high salt products and to accept low salt bread. The manufacture and marketing of traditional bread should be structured and industrial bakeries should be invited to reduce in order to comply with the text being published.

**Funding:** the study was funded by Princess Nourah bint Abdulrahman University Researchers Supporting Project Number (PNURSP2022R43), Princess Nourah bint Abdulrahman University, Riyadh, Saudi Arabia.

### What is known about this topic

- WHO developed the global action plan targeting a 30% reduction of the average salt intake by 2025;
- The consumption of salt in Morocco exceeds the recommendations of the World Health Organization.

### What this study adds

- First study at national level aiming to assess salt content of bread in Morocco;
- Determination of the contribution of bread´s salt in the total salt intake.
